# New hard tick (Acari: Ixodidae) reports and detection of *Rickettsia* in ticks from Sierra Nevada de Santa Marta, Colombia

**DOI:** 10.1007/s10493-023-00887-z

**Published:** 2024-03-14

**Authors:** Ángel Oviedo, Miguel M. Rodríguez, Fernando S. Flores, Lyda R. Castro

**Affiliations:** 1 Grupo de investigación Evolución, Sistemática y Ecología Molecular (GIESEMOL), Universidad del Magdalena, Santa Marta, Colombia; 2 Consejo Nacional de Investigaciones Científicas y Técnicas (CONICET), Instituto de Investigaciones Biológicas y Tecnológicas (IIByT), Córdoba, Argentina; 3 Centro de Investigaciones Entomológicas de Córdoba (CIEC), Facultad de Ciencias Exactas, Físicas y Naturales, Universidad Nacional de Córdoba, Córdoba, Argentina

**Keywords:** *Amblyomma*, *Haemaphysalis*, *Ixodes*, *Rickettsia*, Sierra Nevada de Santa Marta

## Abstract

The Sierra Nevada de Santa Marta (SNSM), located in northern Colombia, is considered a geographical island with high levels of biodiversity and endemism. However, little is known about tick species and their associated microorganisms at the SNSM. In this study we sampled host-seeking ticks in areas of the town of Minca within the SNSM. We collected 47 ticks identified as *Amblyomma pacae*, *Amblyomma longirostre*, *Amblyomma ovale*, *Amblyomma mixtum*, *Haemaphysalis juxtakochi*, *Ixodes* sp. cf. *Ixodes* *affinis* and *Ixodes* sp. Of these ticks, we tested for *Rickettsia* spp. by amplifying the *gltA*, *SCA1*, and 16S rRNA genes via PCR. *Rickettsia amblyommatis* was detected in one pool of 3 larvae and in a female of *A. pacae*. Additonally, we isolated *Rickettsia* sp. belonging to the group of spotted fevers in larvae of *A. longirostre*. This study reports new findings of six species of ticks and two species of *Rickettsia* within the SNSM.

**Supplementary Information:**

The online version contains supplementary material available at 10.1007/s10493-023-00887-z.

## Introduction

Hard ticks (Acari: Ixodida: Ixodidae) are obligate blood-sucking ectoparasites that parasitize the vast majority of vertebrates (Guglielmone et al. 2014). The prevention of tick-borne disease transmission to hosts depends on a thorough understanding of the presence and distribution of ticks, as well as their pathogens (Fritz 2009). This information can only be obtained through sustainable active and passive surveillance, to identify spatial and temporal acarological and epidemiological risk (Han et al.^2021^; Lyons et al. 2021). Without this information, it becomes difficult and inefficient to apply prevention strategies to mitigate the prevalence of ticks and tick-borne pathogens. Furthermore, without the baseline characterization of these metrics, the ability to monitor the effects of climate change or habitat modification on the risk of tick-borne diseases is lost (Johnson et al. 2022).

The Ixodidae are of great relevance for public and veterinary health, since they can cause considerable damage to their hosts such as dermatosis, anemia and even paralysis (Claveria et al. 2022). Additionally, they are vectors of a wide number of parasites including viruses, bacteria, protozoa and nematodes (Guglielmone and Robbins 2018). Infectious agents transmitted by ticks are the cause of important zoonoses that affect humans and domestic animals, among them, the best known zoonoses include Rickettsioses, Borreliosis, Babesiosis, Anaplasmosis, Ehrlichiosis, Q fever and Encephalitis (Jongejan and Uilenberg 2004; Boulanger et al. 2019).

Hard ticks are distributed in all continents, with the highest diversity in tropical regions (Mihalca et al. 2011). Tick establishment in a certain area is dictated by the presence of suitable hosts, optimal climatic and microclimatic conditions and habitat structure. Although ticks are cosmopolitan, they are most abundant in warm climates and tropical regions where oviposition, egg and interstate development are more rapid, and in associations to bushes, shrubs, grasslands and understory (Guglielmone et al. 2004; Labruna 2009; Domínguez et al. 2019; Gilbert 2021; Mathisson et al. 2021). Tick richness in Colombia is represented by 42 species: *Amblyomma* (26), *Ixodes* (10), *Haemaphysalis* (2), *Dermacentor* (2) and *Rhipicephalus* (2) (Rivera-Páez et al. 2018; Ortíz-Giraldo et al. 2021; Benavides-Montaño et al. 2022; Guglielmone et al. 2023). In lower regions of the Sierra Nevada de Santa Marta (SNSM) Ixodid species such as *Amblyomma dissimile*, *Amblyomma mixtum*, *Amblyomma parvum*, *Rhipicephalus sanguineus*, *Rhipicephalus microplus* and *Dermacentor nitens* have been reported associated with domestic animals, amphibians and reptiles (Santodomingo et al. 2018, 2019; Cotes-Perdomo et al. 2020).

On the other hand, in Colombia, different species of *Rickettsia* spp. have been reported, detected in humans, rodents and ticks, including, *Candidatus* Rickettsia asemboensis, *Candidatus* Rickettsia colombianensi, *Candidatus* Rickettsia senegalensis, *Candidatus* Rickettsia tarasevichiae, *Rickettsia amblyommatis*, *Rickettsia bellii*, *Rickettsia canadensis*, *Rickettsia felis*, *Rickettsia monacensis*, *Rickettsia parkeri*, *Rickettsia prowazekii*, *Rickettsia rickettsii*, *Rickettsia rhipicephali *and *Rickettsia tamurae* (Quintero et al. 2013, 2017; Miranda and Mattar 2014; Faccini-Martínez et al. 2015, 2016; Cardona-Romero et al. 2020; Miranda et al. 2020; Martínez-Sánchez et al. 2021; Díaz et al. 2023). Within the SNSM there are reports of *Rickettsia* species in Guajira, Magdalena and Cesar regions, specifically of *Candidatus* R. colombianensi, *R. rhipicephali*, *R. monacensis* and *R. belli*, detected in ticks collected in domestic animals, reptiles and birds (Santodomingo et al. 2018, 2019; Cotes-Perdomo et al. 2020; Rodriguez et al. 2023). However, these reports have only been made in tropical dry forest ecosystems and at altitudes below 600 m above sea level (masl). Despite *Babesia, Anaplasma* and *Ehrlichia* have also been detected from ticks and domestic animals in the area, there are still few studies investigating the presence and distribution of ticks in the Caribbean, including the SNSM, despite its potential epidemiological importance (Cotes-Perdomo et al. 2018, 2020; Santodomingo et al. 2018, 2019; Pesapane et al. 2019).

Minca, with an altitude between 600 and 2000 masl, is considered the ecological capital of the SNSM due to its high level of biodiversity, recognized for its scientific and ecological tourism, it has a high flow of tourists, the vast majority of whom are of international origin (Mayor’s Bulletin 2022a, b; WRadio Bulletin 2023). As a highly touristic destination, it could be a gateway for the exit or entry of new pathogens, enabling their establishment in the area, making it vulnerable to economic, ecological and social repercussions (Hall 2019). In general, in Colombia, eco-epidemiological tick studies are diverse, but they have been focused on areas where outbreaks have occurred, leaving a void in areas with epidemiological silence. Therefore, there is a latent need to identify and know the possible vectors and pathogens to which wild and domestic animals and humans could be exposed. The present study aims to report the presence of ticks and *Rickettsia* spp. collected in Minca, a touristic town located at approximately at 1000 masl, at the SNSM, Colombia.

## Materials and methods

### Sample collection

Ticks were collected in three places at the Minca region, located in the foothills of the SNSM through sampling in the months of February and November 2022. The SNSM is a mountainous massif that is isolated from the Andes. Its highest peak rises to 5776 masl and its total area is of approximately 17,000 km^2^ (Huertas-Díaz et al. 2017). The SNSM presents variations in climate and vegetation produced by altitude (Rangel and Garzon 1995), however this region presents rapid changes in the plant formations depending on the geographic position, from dry deciduous and arid succulent shrubs to humid evergreen seasonal forest. Therefore, it has been established an altitudinal zonation with different biomes such as Lowland Xerophytic scrub forest (0-250 m), Low montane wet forest (0-600 m), Cloud Forest (700-1800 m), Andean Forest (2300-3200 m), Paramo (3300-4400 m) and Superparamo (4500 m-snow limit) (Bernal-Carlo 1991). The variety of climates and biomes that the SNSM presents, provide ecosystems with high levels of endemism and biological diversity (Camero 2002).

**Fig. 1:**
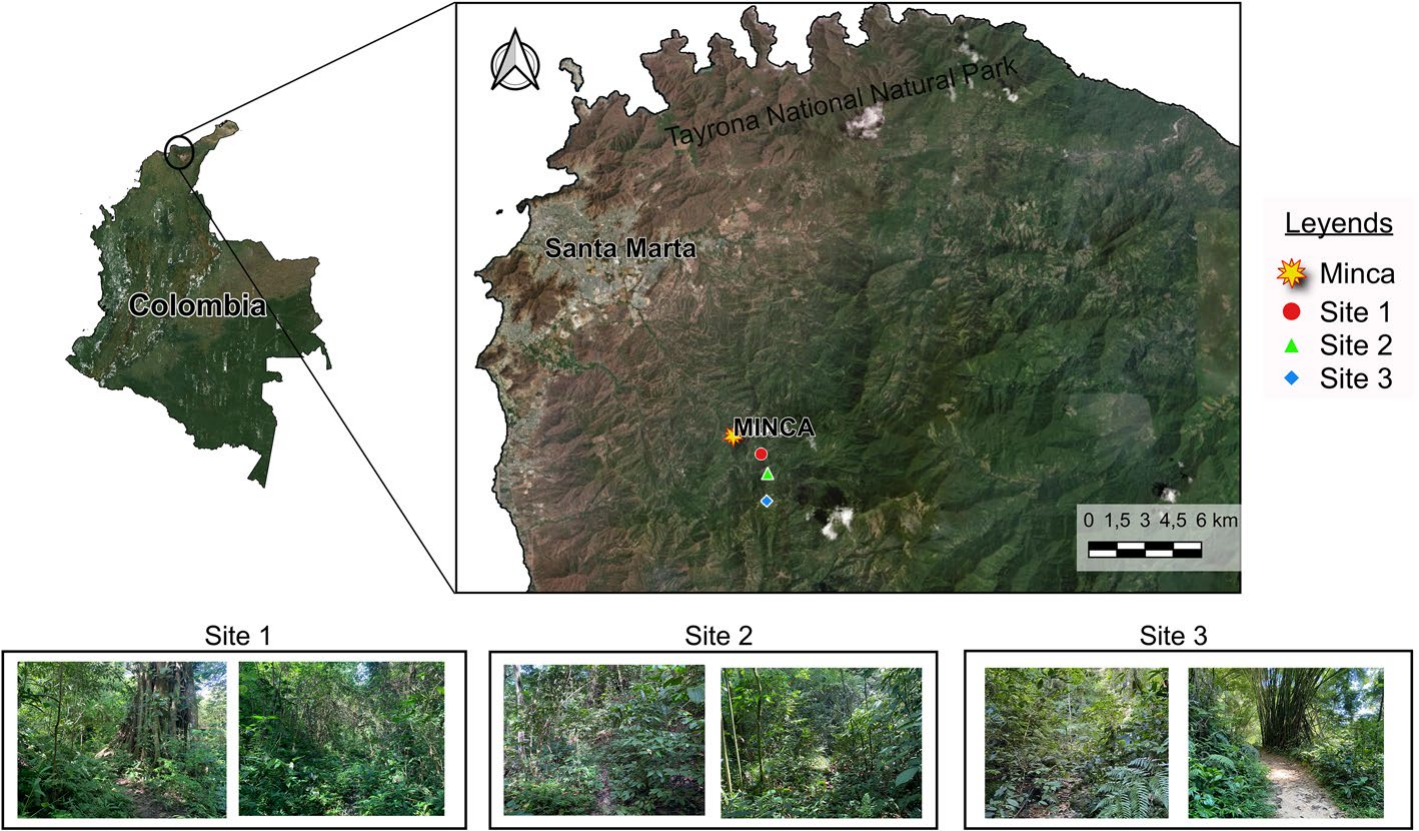
Map of the geographic location of the sampling sites at Minca, Sierra Nevada de Santa Marta, Colombia

The first sampling site (11°08’02.8’’N, 74°06’07.5’’W) is located at an altitude of 800 masl, the second site (11°7’1.48"N, 74°5’51.78"W) is located at an altitude of 1200 masl, while the third site (11°06’42.1"N, 74°05’49.5"W) is at 1296 masl (Fig. 1). All places have Cloud forests, characterized by dense, hygrophytic and sub-hygrophytic vegetation, highlighting the presence of trees, tree ferns, mosses, liverworts, lichens and bamboos, being Melastomataceae, Piperaceae, Moraceae, Bromeliaceae, *Solanum, Pleurothallis, Passiflora, Polypodium, Psychotria, Bambusa, Coenogonium, Notothylas, Radula, Cladonia, Hypotrachyna* and *Stereocaulon* the predominant families and genera (Rangel and Garzón 1995; Bernal 2016; Gradstein et al. 2016). Additionally, sampling localities have a rainfall regime of 3000 mm per year and have frequent fogs that increase humidity (Adams 1973; Rangel and Carvajal-Cogollo 2012). Tick collection was carried out by using the dragging and flagging methods, in which a 1 m^2^ piece of cotton cloth was swept over the vegetation near human and animal trails (Salomon et al. 2020). At each site, the collection was carried out by four operators for 1.30 h each, in the month of February and in the month of November 2022, the distance between sites one and two is of 3.8 km and the distance between sites two and three is of 3.5 km. Ticks on the cloth were removed with fine-tipped forceps and preserved in 99% ethanol.

### Taxonomic identification of ticks

Morphological identification of *Amblyomma* adults and nymphs was made following Martins et al. (2010) and Nava et al. (2017) and the larvae according to Barros-Battesti et al. (2005) and Barbieri et al. (2008a, b). A nymph of *Haemaphysalis* was identified following Nava et al. (2017). The adults of *Ixodes* were identified following Nava et al. (2017) and Saracho-Bottero et al. (2020), while the nymphs and larvae following Sénevet and Ripert (1967) and Durden and Keirans (1994). After identification, the specimens were discriminated by species and stage for storage.

### Molecular identification of ticks

DNA extraction was performed using the MasterPure DNA purification kit (Lucigen, USA). Depending on the numbers of ticks collected per species, only a subset of the collected ticks was taken at random for the molecular analyzes. As the majority of the tick species found had not been reported for the study region, it was important to leave ticks for a possible detailed morphological description and biological collections. Adult stage ticks and nymphs were cut transversally with a sterile scalpel in the area of the festoons for the lysis process and to preserve the body structure. Larvae were processed individually or in pools (the pools consisted of a maximum of 10 larvae, depending to the number of individuals collected) and macerated with sterile pestles. All samples were incubated at 37 °C overnight. DNA quality was verified through 1% agarose gel electrophoresis, using RedGel (Biotum). Subsequently, the mitochondrial gene cytochrome c oxidase subunit I (*cox1*) and the 16S rDNA gene, also mitochondrial, were amplified by conventional PCR. For the *cox1* amplification, we used the primers LCO1490 (5’-GGTCAACAAATCATAAAGATATTGG-3’) and HCO2198 (5’-TAAACTTCAGGGTGACCAAAAATCA-3’) described by Folmer et al. (1994), generating a 658 bp fragment. A second pair of primers, ArF2 (GCICCIGAYATRGCITTYCCICG) and ArR5 (CCIGTIYTIGCIGGIGCI ATYAC) described by Gibson et al. (2014) was also used. For the 16S rDNA, we used the primers 16S + 1 (5’-CCGGTCTGAACTCAGATCAAGT-3’) and 16S-1 (5’-GCTCAATGATTTTTAAATTGCTGT-3’) described by Mangold et al. (1998), obtaining a 460 bp fragment.

The amplifications were performed in a volume of 25 µl with: 0.5 µl of Taq polymerase (5 U/µl, Bioline, EE. UU.), 2.5 µl of PCR buffer (10X, Bioline, EE. UU.), 1 µl of MgCl (50 mM, Bioline, EE. UU.), 1 µl of dNTPs (10 Mm, Bioline, EE. UU.), 1 µl of each primer (10 pmol), 15 µl de ddH2O and 3 µl of DNA. Amplification conditions were as follows: initial denaturation at 94 °C for 3 min, followed by 35 cycles at 94 °C for 30 s, alignment at 45 °C (*cox1) /* 56 °C (16S rRNA) for 30 s, extension at 72 °C for 1 min, and a final extension at 72 °C for 7 min.

### Detection of Rickettsia

A total of 14 samples (4 adults, 4 nymphs, 3 larvae and 3 pools of larvae) were tested for the presence of *Rickettsia*. To determine the presence of *Rickettsia* spp. in the DNA of the collected samples, the *gltA* gene was amplified by conventional PCR, using the primers CS-78 (5’-GCAAGTATCGGTGAGGATGTAAT-3’) and CS-323 (5’-GCTTCCTTAAAATTCAATAAATCAGGAT-3’) described by Labruna et al. (2004b, c), generating a 401 bp fragment. To corroborate the presence of *Rickettsia* spp. and obtain a clear identification, the samples with a positive result from the *gltA* gene, were also targeted the 16S rRNA and *SCA1* genes amplification. The 16S rRNA gene was amplified using the primers Rick-16Ss-F3 (5′-ATCAGTACGGAATAACTTTTA-3′) and Rick-16 S-R4 (5′-TGCCTCTTGCGTTAGCTCAC-3′), generating a 1332 bp amplicon. The *SCA1* gene was amplified using the primers SCA1-F2 (5′-GGTGATGAAGAAGAGTCTC-3’) and SCA1-R2 (5′-CTCTTTAAAAATGTTCTACATT- 3’), generating a 488 bp fragment, both described by Anstead and Chilton (2013).

*Rickettsia* amplifications were performed in a volume of 25 µl with: 0.5 µl of Taq polymerase (5 U/µl, Bioline, EE. UU.), 2.5 µl of PCR buffer (10X, Bioline, EE. UU.), 1 µl of MgCl (50 mM, Bioline, EE. UU.), 1 µl of dNTPs (10 Mm, Bioline, EE. UU.), 1 µl of each primer (10 pmol), 15 µl de ddH_2_O and 3 µl of DNA. Amplification conditions were as follows: initial denaturation at 94 °C for 3 min, followed by 40 cycles at 94 °C for 30 s, alignment at 48 °C (*gltA)*/49°C (*SCA1)*/58°C (16S rRNA) for 30 s, extension at 72 °C for 2 min, and a final extension at 72 °C for 7 min. All PCRs included positive and negative controls, the positive control for rickettsia were positive DNAs obtained and sequenced in previous studies (Rodriguez et al. 2023). PCRs were verified through 2% agarose gel electrophoresis, stained with RedGel (Biotum). Positive samples were cleaned and sequenced by Sanger technology in both directions at SSigMol, the sequencing service from Universidad Nacional.

### Phylogenetic analysis

Sequence files were edited using the Biological Sequence Alignment Editor (BioEdit v7.2.5) (Hall 1999). Subsequently, they were subjected to a similarity analysis with sequences from the NCBI database (www.ncbi.nlm.nih.gov), using the nBLAST tool (Altschul et al. 1990). As a general rule, we used a species (query sequence) percentage of 97% or greater similarity to be assigned to same species (Stackebrandt and Goebel 1994; Hebert et al. 2003; Chung et al. 2018). For the coding gene sequences, the reading frame was corrected using the Aliview software (Larsson 2014) and a data matrix was prepared with reference sequences from Genbank, which were aligned by codons and manually curated in Geneious prime (Kearse et al. 2012). Non-coding genes were aligned by nucleotides with other reference sequences and cured with Gblocks 0.91b. All data sets were aligned using the Mafft algorithm (Katoh et al. 2002). We used the PartitionFinder2 software (Lanfear et al. 2012), applying the Bayesian Inference Criterion (BIC) to find the best substitution models. Maximum Likelihood Phylogenetic analyzes were performed on IQ-TREE (Nguyen et al. 2015), using the Bootstrap algorithm with fast search and using 10,000 pseudo replicates. Also, phylogenetic trees based on Bayesian Inference were run in MrBayes (Ronquist et al. 2012), implementing 5,000,000 generations, with trees sampled every 100 generations, discarding 25% of the trees. Nodes with Bootstrap values and posterior probability greater than > 70% indicated high statistical support (Hillis and Bull 1993).

## Results

### Ticks species

**Fig. 2:**
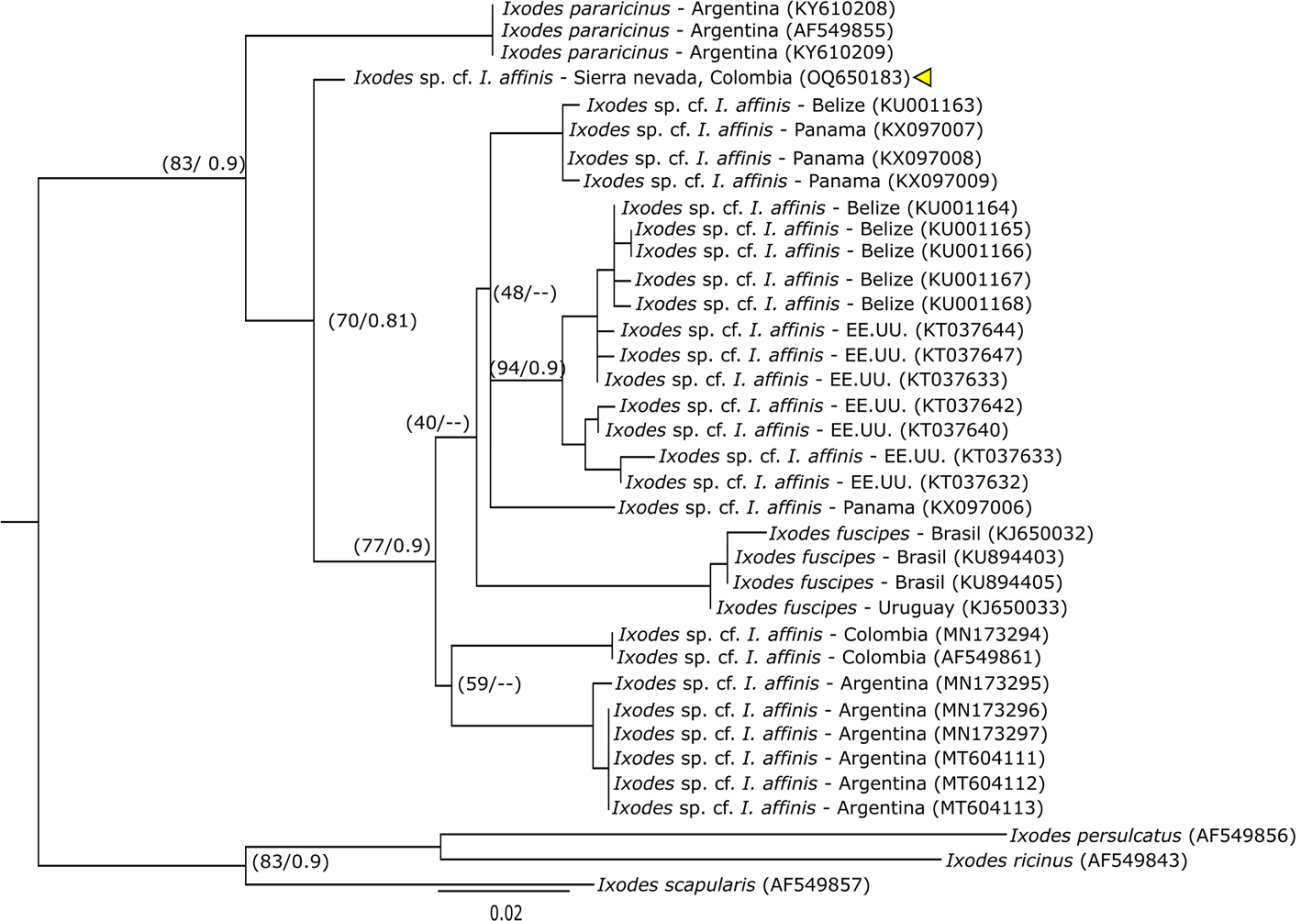
Phylogenetic reconstruction using maximum likelihood and Bayesian inference of 16S rDNA for our sequence (yellow triangle) and sequences downloaded from GenBank of the *Ixodes ricinus* complex (outgroup). Numbers on nodes correspond to bootstrap values/the posterior probability. (Color figure online)

**Fig. 3:**
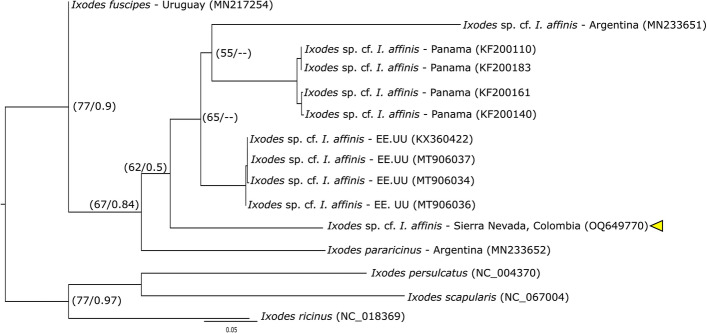
Phylogenetic reconstruction using maximum likelihood and Bayesian inference of *cox1* for our sequence (yellow triangle) and sequences downloaded from GenBank of *Ixodes ricinus* complex (outgroup). Numbers on nodes correspond to bootstrap values/the posterior probability. (Color figure online)

**Fig. 4:**
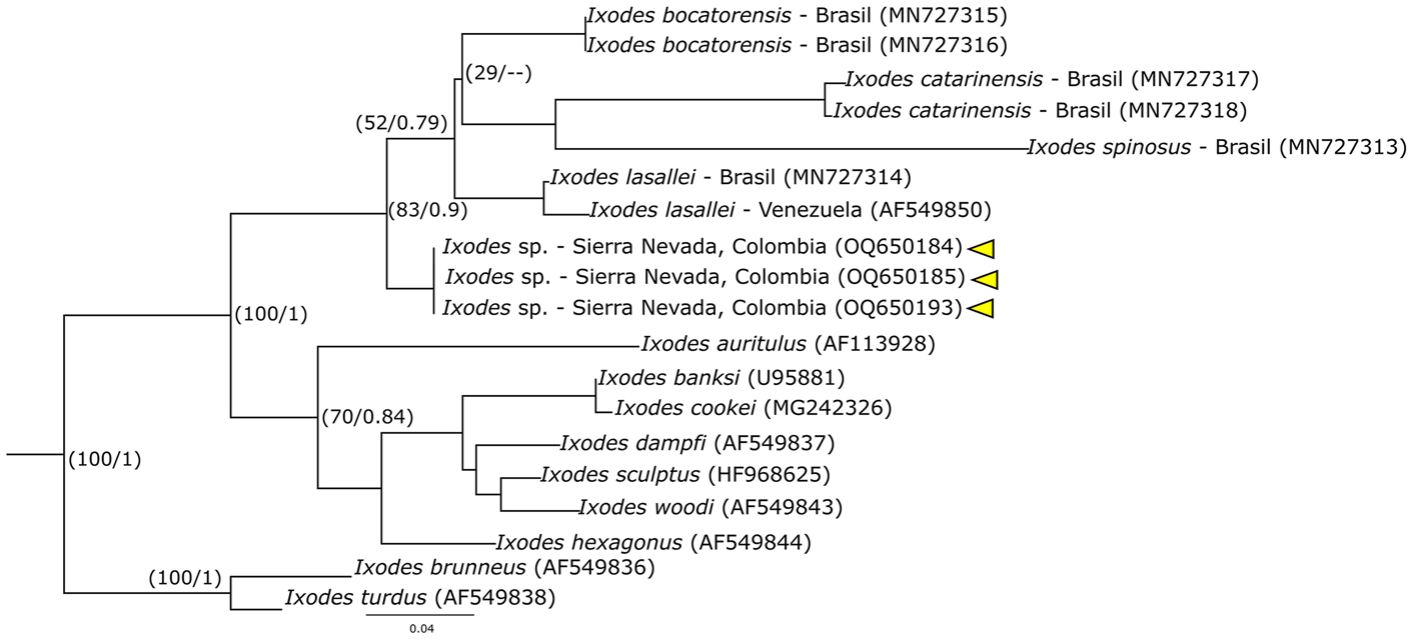
Phylogenetic reconstruction using maximum likelihood and Bayesian inference of 16S rDNA for our sequences (yellow triangle) and sequences downloaded from GenBank of tick species of the genus *Ixodes*. Numbers on nodes correspond to bootstrap values/the posterior probability. (Color figure online)

A total of 47 ticks (35 larvae, 4 nymphs and 8 adults) were collected and identified, distributed in three genera of the Ixodidae family: *Ixodes* (2 species), *Amblyomm*a (4 species) and *Haemaphysalis* (1 species) (Table 1). For *Ixodes*, the sequence of one male, morphologically identified as *Ixodes* sp. cf. *I. affinis* (Fig. S1), presented similarly values of 96.35% for the 16S rDNA gene (KT037640) and 91.55% for the *cox1* gene (KX360422) with sequences corresponding to *Ixodes* sp. cf. *I*. *affinis*. The analysis of Maximum Likelihood and Bayesian Inference of the 16S rDNA gene (Fig. 2), placed the sequence of *Ixodes* sp. cf. *I. affinis* within the *Ixodes ricinus* complex, but outside the clade made up of *Ixodes fuscipes* and four lineages of *Ixodes* sp. cf. *I. affinis* with reference sequences from Argentina, Panama, Colombia, the United States and Belize. Similar results were obtained using the *cox1* gene sequences (Fig. 3). On the other hand, sequences from one larva and two nymphs identified as *Ixodes* sp. (Figs. S2, S3), presented a Blast similarity of 93.29% with sequences of *Ixodes lasallei* (MN727314) for the 16S gene, and of 82.35% with sequences from *Ixodes dentatus* (KX360374) for the *cox1* gene, given that there are no sequences of the *cox1* gene for *I. lasallei* in GenBank. In the phylogenetic analyzes, these sequences were closely related to the monophyletic clade made up of *I. lasallei*, *Ixodes bocatorensis*, *Ixodes catarinensis* and *Ixodes spinosus*, which does not indicate a clear identification of the specimens (Fig. 4).

A total of 42 ticks were taxonomically identified as *Amblyomma*: *Amblyomma longirostre* (29 larvae), *Amblyomma ovale* (1 larva, 1 female and 4 males), *Amblyomma pacae* (3 larvae and 1 female) and *A. mixtum* (2 nymphs and 1 male). *Amblyomma longirostre* sequences obtained presented 100% identity with *A. longirostre* sequences corresponding to the 16S (MH818419) and the *cox1* (MT180842) genes (Fig. S4). Sequences obtained from the female and one larva of *A. pacae* yielded a similarity percentage of 100% (KU001159; KY020985) for the 16S gene and of 97.36-99.09% for the *cox1* gene with sequences corresponding to *A. pacae* (MH513236) (Fig. S4). The sequences obtained for *A. ovale*, presented a similarity of 99.76% with *A. ovale* sequences for the 16S gene (MN557258.1) and 99.48% for the *cox1* gene (ON134096.1) (Fig. S4). For *A. mixtum*, the sequences obtained had a 98.72-99.75% (KT820359, MG938670) similarity for the 16S and 99.69-100% (MT549811, KF200097) for *cox1* with sequences from *A. mixtum* (Fig. S4). Finally, a nymph of *H. juxtakochi* presented an identity of 98.08% (MH513302) for the 16S gene and 99.85% (KF200092) for the *cox1* gene, with reference to *H. juxtakochi* sequences (Fig. S4).

**Table 1:** Molecular detection of *Rickettsia* in host-seeking Ixodidae ticks collected from Sierra Nevada de Santa Marta, Colombia. Including GenBank accession numbers of the sequences obtained. L = Larva, N = Nymph, M = male, F = Female

Site	Tick species	No. of ticks collected	No. of sequences for gene (16 S/cox1)	Tick deposited sequences (16 S/cox1)	Presence of Rickettsia	Rickettsia species	No. of sequences for gene (gltA/SCA1/16S)	Rickettsia deposited sequences (gltA, SCA1, 16 S)
1	*Amblyomma ovale*	1 M/1F						
	*Amblyomma mixtum*	1 M	1	OQ650182/ OQ649769				
2	*Amblyomma ovale*	2 M	1	OQ650189/ OQ649775				
	*Amblyomma pacae*	3 L/1F	2	OQ650186, OQ650187/ OQ645681, OQ649773	1 pool 1 F	*Rickettsia amblyommatis*	2	OQ656319/ OQ675543/ OQ674713, OQ656320/ OQ675544/ OQ674714
	*Amblyomma mixtum*	2 N	2	OQ650188, OQ650192/ OQ649774, OQ649778				
3	*Ixodes* sp. cf. *I. affinis*	1 M	1	OQ650183/ OQ649770				
	*Ixodes* sp.	1 L/2 N	3	OQ650184, OQ650185, OQ650193/ OQ649771, OQ649772, OQ649779				
	*Amblyomma ovale*	1 L/1 M	1	OQ650195/ OQ649780				
	*Amblyomma longirostre*	29 L	2	OQ650190, OQ650194/ OQ649776, OQ645682	1 pool (10 larvae)	*Rickettsia* sp.	1	OQ656321/ OQ675545/ OQ674715
	*Haemaphysalis juxtakochi*	1 N	1	OQ650191/ OQ649777				

### Detection of Rickettsia

**Fig. 5:**
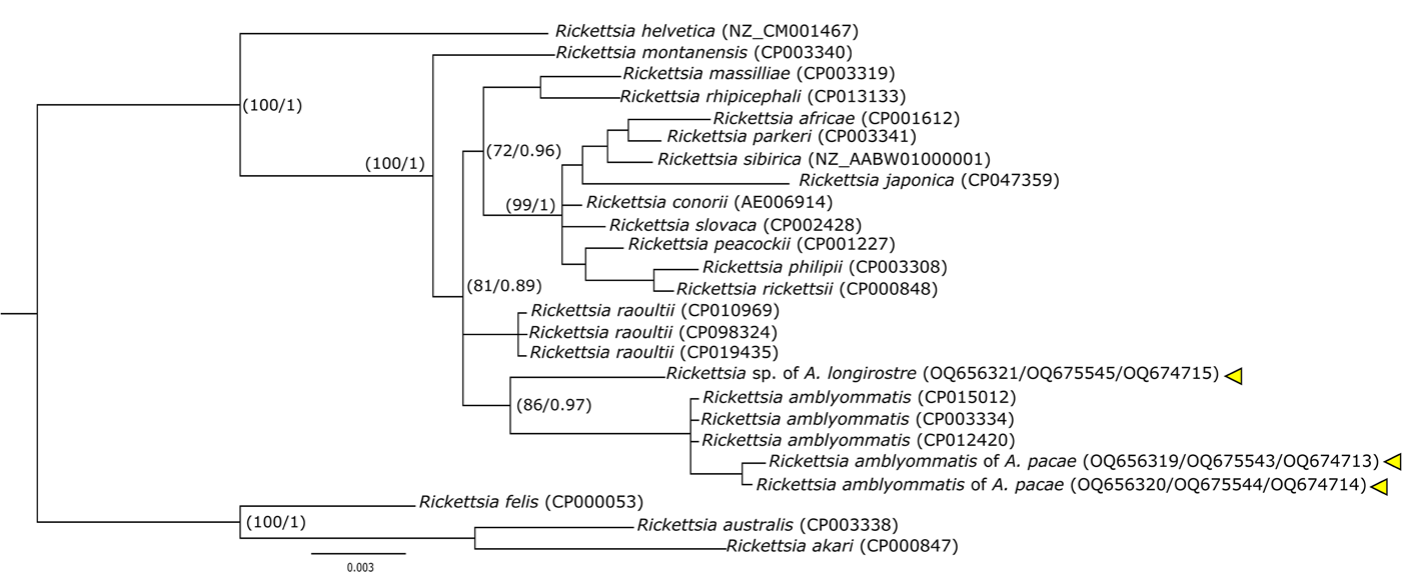
Phylogenetic reconstruction using maximum likelihood and Bayesian inference of concatenate *gltA*, *SCA1* and 16S rRNA genes for genus *Rickettsia*, including our sequences (yellow triangle) and sequences downloaded from genbank. Numbers on nodes correspond to bootstrap values/the posterior probability. (Color figure online)

We obtained positivity for *Rickettsia* in *A. pacae* (1 female and 1 pool of three larvae) and *A. longirostre* (1 pool of ten larvae) (Table 1). The obtained sequences in *A*. *pacae* showed an identity of 100% for *gltA* (MN947702), 99.68% for *SCA1* (CP015012) and 99.83–100% for 16S rRNA (CP015012) with *R. amblyommatis*. Similarly, the Maximum Likelihood analysis and Bayesian Inference grouped the sequences obtained with *R. amblyommatis* sequences (Fig. 5). In *A. longirostre*, the sequences obtained showed a 99% identity for *gltA* (MN947699), 99.83% for 16S rRNA (MK304546) with *Rickettsia raoultii* and a 99.35% similarity for *SCA1* with *R. amblyommatis* (CP015012). However, in Maximum Likelihood and Bayesian Inference analyzes, this sequence forms a clade with *R. amblyommatis*, which is closely related to *R. raoultii* (Fig. 5). Since there is no clear identification for the sequence obtained from this pool, it was categorized at the genus level as *Rickettsia* sp.

## Discussion

For the SNSM and the Magdalena region there were no previous records for ticks of the genera *Ixodes* and *Haemaphysalis*, despite the fact that several species of these genera have been reported in other areas in Colombia. Therefore, this is the first report in this area of two species of the genus *Ixodes* (*Ixodes* sp. cf. *I*. *affinis* and *Ixodes* sp.), one species of the genus *Haemaphysalis* (*H. juxtakochi*) and two species of the genus *Amblyomma* (*A. pacae* and *A. longirostre*) (Acevedo-Gutiérrez et al. 2020; Uribe et al. 2020).

*Ixodes* sp. cf. *Ixodes affinis* presents problems in their morphological identification, since Rodríguez-Vivas et al. (2016) found differences in the shield scores of *Ixodes affinis* specimens as compared to the original description by Neumann, 1899. Nadolny et al. (2016) found genetic differences in 16S rDNA sequences from specimens from Colombia, Panama, Belize and the United States, forming independent clades according to the geographic location. This was supported by Saracho-Bottero et al. (2020), when analyzing sequences of specimens from Argentina, Belize, Colombia, the United States and Panama. They also found differences according to geographic location and defined the *I*. *affinis* specimens as a complex made up of four lineages, leaving them in the taxonomic status of *Ixodes* sp. cf. *I*. *affinis*.

The male of *Ixodes* spp. analyzed in this study (Fig. S1) presented morphological similarity with ticks belonging to the *I. affinis* complex, structures such as scutal punctuations and the length of the posterior lateral denticles of the hypostome were compatible with *Ixodes* sp. cf. *I*. *affinis* from Argentina (Saracho-Bottero et al. 2020). In phylogenetic analyses the sequence was closely related to the *I*. *affinis* complex but unrelated from the sequences for *Ixodes* sp. cf. *I*. *affinis* previously detected in Colombia (Saracho-Bottero et al. 2020) and did not cluster with any of the known lineages for this complex. Therefore, further study is required to determine whether the *Ixodes* sp. cf. *I. affinis* from the SNSM corresponds to a new lineage of the *I*. *affinis* complex.

Ticks of the *I. affinis* complex have been found parasitizing a wide variety of birds and mammals, such as bovines, canines, felids, equines, procyonids, didelphids, cricetids and ursids (Guzmán-Cornejo et al. 2007; Harrison et al. 2010; Bermúdez et al. 2015; Heller et al. 2016; Rodríguez-Vivas et al. 2016; Flores et al. 2023a). The bird diversity at the SNSM is comprised of more than 600 species of birds (Rodríguez-Navarro 2000), the passerine order being the most abundant. There are 14 endemic species of birds to this ecosystem. Regarding terrestrial mammals, there is a lack of information on the true diversity, however, there are records of species from the family Cervidae, Felidae, Mustelinidae, Canidae, Dasypodidae, Didelphidae, Cuniculidae, Dasyproctidae, Heteromidae, Sciuridae and Erethizonthidae (Granados-Peña et al. 2014; Pineda-Guerrero et al. 2015).

*Ixodes affinis* has veterinary medical importance since they are potential vectors of *Borrelia burgdorferi* sensu lato (Harrison et al. 2010; Maggi et al. 2010; Heller et al. 2016; Flores et al. 2020). In Colombia, there are reports indicating the presence of *Borrelia* species from the relapsing fever group and Lyme disease group, including *B. burgdorferi* sensu stricto in bats and rodents in the southwestern regions of the country (Mancilla-Agrono et al. 2022). Thus, future studies should focus in evaluating the presence of *Borrelia* in the *Ixodes* ticks of the SNSM.

One larva and two nymphs (Fig. S2, S3) were morphologically identified as *Ixodes* sp., as descriptions of these stages are currently lacking for many species of the genus *Ixodes* (Guglielmone et al. 2021). However, with the phylogenetic analysis of the 16S gene, it was possible to determine that the 3 specimens belong to the same taxa, which was grouped in the clade formed by *I. bocatorensis*, *I. catarinensis, I. lasallei* and *I. spinosus*. Adults of *I. bocatoriensis* and *I. lasallei* were found by Apanaskevich and Bermúdez (2017) in Colombia in the regions of Antioquia and Arauca, respectively. Although there are few records, the adults of these species have been found associated mainly with rodents of the Cunicululidae and Dasyproctidae families (Guglielmone et al. 2021). These mammals have been found in the sampled area (Díaz et al. 1986; Pineda-Guerrero et al. 2015), so future studies should be carried out to find adults of this tick species and to be able to make the corresponding morphological comparisons. It is important to highlight that it could be *Ixodes venezuelensis*, a morphologically similar species (Durden and Keirans 1994) and for which there is no genetic information.

*Haemaphysalis juxtakochi* is a species widely distributed in the Nearctic and Neotropical zones of America (Guglielmone et al. 2014), however the only record in Colombia was made by Kohls (1960) on *Mazama* sp. and *Tapirus* sp. in the Meta region, more than 60 years ago. The primary hosts of *H. juxtakochi* are Artiodactyl mammals belonging to the family Cervidae (Jones et al. 1972; Martins et al. 2007). For the SNSM there are records of species of brocket deer such as *Mazama americana*, *Mazama gouazoubira* (Díaz et al. 1986) and *Mazama sanctaemartae* (Granados-Peña et al. 2014). Additionally, *H. juxtakochi* has been reported on mammals, birds and humans (Beldomenico et al. 2003; Venzal et al. 2005; Bermúdez et al. 2012; Nava et al. 2017, Flores et al. 2023a) in South America. This tick has been found infected with *R. rhipicephali* (Labruna et al. 2007), *R*. *amblyommatis* (Castro et al. 2015), *R*. *parkeri* (Souza et al. 2018) and *Candidatus* Ehrlichia pampeana (Felix et al. 2021; Flores et al. 2023b).

*Amblyomm*a *longirostre* is a Neotropical tick, with reports from Mexico to Argentina (Nava et al. 2010; Guglielmone et al. 2023). For Colombia, the presence of *A*. *longirostre* has been reported for the regions of Boyacá (Osorno-Mesa 1940), Meta (Wells et al. 1981), Arauca (Cardona-Romero et al. 2020) and Caldas (Martínez-Sánchez et al. 2021), parasitizing *Coendou prehensilis* (known as porcupine), *Coendou* sp. and birds. Adults of *A*. *longirostre* are usually found on rodents of the family Erethizontidae, while the larvae and nymphs feed on different families of birds (Guglielmone et al. 2021). A species of porcupine of the family Erethizontidae (*C. prehensilis*) that lives at the SNSM (Ramírez-Chaves et al. 2016), has been found with *A*. *longirostre* (Aragão 1918; Labruna et al. 2002), so it could be a common host supporting the population in this area. Regarding the epidemiological importance of *A*. *longirostre*, they are known to feed on humans (Arzua et al. 2005; Rodríguez-Peraza et al. 2014) and have shown to be positive for *R*. *amblyommatis* (Labruna et al. 2004b, c; Ogrzewalska et al. 2008; Pacheco et al. 2012) and *R*. *belli* (McIntosh et al. 2015). The *Rickettsia* sp. detected in this study grouped within the spotted fever group *Rickettsia* and was closely related to *R. amblyommatis* and *R. raoultii*, which requires further study to obtain a clear identification of this bacterium. A limitation in our study is that this positive was detected in a pool of larvae of 10 individuals, therefore the low sequence similarity may be due to multiple larvae infected with different species of *Rickettsia*. Thus, without extracting and testing the individual larvae we cannot discern this finding.

*Amblyomma ovale* has a wide distribution in the Neartic and Neotropical region, finding records from the United States to Argentina (Guglielmone et al. 2023). In Colombia, there are previous reports in Antioquia, Caldas, Chocó, Córdoba, Cundinamarca, Guaviare, Meta, Nariño, Sucre, Tolima and Valle del Cauca (Paternina et al. 2009; Londoño et al. 2017; Rivera-Páez et al. 2018; Acevedo-Gutiérrez et al. 2020; Uribe et al. 2020; Martínez-Sánchez et al. 2021). Adults of *A. ovale* feed mainly on Carnivora and Perisodactyla (wild and domestic), and immature stages on rodents, marsupials and birds (Martins et al. 2012; Guglielmone et al. 2021). This species of tick has medical-veterinary importance since it is a frequent parasite of domestic animals and humans, with human records in different parts of the Neotropical region (Labruna et al. 2004b, c; Nava et al. 2007; Guzmán-Cornejo et al. 2011; Quintero et al. 2017) including the SNSM (personal communication). In addition, *R*. *parkeri* and *R*. *belli* were detected in *A.* *ovale *from several countries (Labruna et al. 2004b, c; Szabó et al. 2013; Lamattina et al. 2018; Sánchez-Montes et al. 2019), including Colombia (Londoño et al. 2014) and *Ehrlichia* sp. in Argentina (Tarragona et al. 2022).

*Amblyomma mixtum* is a generalist tick, reported on various domestic and wild vertebrates, including orders such as Amphibia, Aves, Mammalia and Reptilia (Guglielmone et al. 2021), distributed in southern Texas, Central America, the Caribbean islands, Colombia and Ecuador (Beati et al. 2013; Rivera-Páez et al. 2016). *Amblyomma mixtum* belongs to the *Amblyomma cajennense* complex made up of *Amblyomma patinoi*, *Amblyomma tonelliae*, *Amblyomma interandinum*, *Amblyomma sculptum* and *Amblyomma cajennense* s.s., clearly differentiated at the molecular level (Cotes-Perdomo et al. 2023). The ticks of this complex are of great medical-veterinary interest, since they are vectors of *R. rickettsii* in Central and South America (Nava et al. 2014). Some of the *Rickettsia* species that have been detected in *A. mixtum* include: *R. rickettsii* (Bermúdez et al. 2016), *R*. *amblyommatis* (Castro et al. 2015; Bermúdez et al. 2021a), *R*. *typhi* (Ulloa-García et al. 2020), *Ca.* R. colombianensi and *R. rhipicephali* (Santodomingo et al. 2019). In Colombia, *A. mixtum* has been reported in the regions of Antioquia, Arauca, Caldas, Casanare, Córdoba and Meta (Rivera-Páez et al. 2016, 2018; Acevedo-Gutiérrez et al. 2021). For the Magdalena region, Santodomingo et al. (2019) and Cotes-Perdomo et al. (2020), made reports of *A. mixtum* in domestic animals mainly in tropical dry forest matrices and at altitudes below 700 masl. This study reports the presence of *A. mixtum* at altitudes of 1000 masl at the SNSM.

*Amblyomma pacae* is distributed from Paraguay to Mexico (Guglielmone et al. 2021), however the only reports in Colombia correspond to adults collected on *Cuniculus paca* (lowland paca) in the Boyaca and Meta regions, more than 50 years ago (Osorno-Mesa 1940; Wells et al. 1981). The main host is *C. paca* (Jones et al. 1972), a widely distributed species that is present in the SNSM (Pineda-Guerrero et al. 2015). The epidemiological importance of *A. pacae* is unknown, however, in this study infection by *R. amblyommatis* was detected, which adds to the detections of this bacteria in this tick species in Belize and Panama (Lopes et al. 2016; Bermúdez et al. 2021b). *Rickettsia amblyommatis* has been detected in most genera of the Ixodidae family and there are also reports of its presence in Central America, South America and in North America (Labruna and Mattar 2004; Karpathy et al. 2016; Bermúdez and Troyo 2018; Bermúdez et al. 2021a). There are previous reports for the northern part of Colombia, such as the one carried out by Quintero et al. (2017) detecting *R. amblyommatis* in a nymph of *Amblyomma varium* parasitizing a person in the rural areas of Urabá (Antioquia, Colombia) and by seropositivity with IgG antibodies for *R. amblyommatis* in rural workers, *Equus caballus*, *Equus asinus* and *Canis familiaris*. Likewise, Quintero et al. (2020) reported the infection of *R. amblyommatis* in two nymphs of *A. patinoi* collected from humans for the same geographic area.

This study reports for the first time, for Minca at the SNSM, the tick species *A. pacae*, *A. longirostre*, *A. ovale*, *A. mixtum*, *H. juxtakochi*, *Ixodes* sp. cf. *I*. *affinis* and *Ixodes* sp. Likewise, the presence of *R. amblyommatis* is reported in larvae and in a female of *A. pacae* and *Rickettsia* sp. belonging to the group of spotted fevers in *A. longirostre* larvae. Bioclimatic conditions can modulate or become barriers to the distribution of ticks; Clarke-Crespo et al. (2020) in their work on niche modeling in Ixodidae ticks in Mexico, showed that ticks of some genera of *Amblyomma* concentrate at altitudes of 1000 to 1500 masl, because these ticks have low resistance to desiccation and little tolerance to temperature changes. Likewise, Ferrell et al. (2017), only report the presence of ticks such as *A. ovale* and *Ixodes boliviensis* at elevations above 1000 masl, in their work on altitudinal gradient in ticks associated with dogs. However, species such as *A. mixtum* do not tolerate low temperatures, therefore, altitudinal gradients are an ecological barrier that limit their distribution (Aguilar-Domínguez et al. 2021). This correlates to the existing reports for the SNSM, as at lower elevations only species such as *A. mixtum*, *A. dissimile* and *A. ovale* had been reported. However, in this study, we record the presence of *Ixodes* tick species at elevations above 1000 masl.

In this study, site 3 presented a greater diversity, probably because it was a relic of cloud forest in a good state of conservation and a denser vegetation. On the other hand, site 2 was located in a matrix of coffee plantations and site 1 in an area with high tourist impact. These characteristics are not mentioned in the article because it was not our objective to evaluate the conservation status of the sites. The sites were chosen because they were located at altitudes above 700 masl, with entrance permits and the presence of trails that would facilitate the transit of wildlife. This study shows that the tick fauna in the SNSM is still unknown, therefore, more studies are needed to expand the information on the Ixodidae fauna, its hosts and tick-borne pathogens in this region. This information is of public and veterinary health importance because these ticks have been reported as vectors of pathogens, both of veterinary and public health concern, and because the study area (Minca, Santa Marta, Colombia), is recognized for its ecotourism, receiving thousands of national and international tourists each year.

### Supplementary Information

Below is the link to the electronic supplementary material.

Supplementary material 1 (DOCX 2980.2 kb)
