# Supplementary material for: New hard tick (Acari: Ixodidae) reports and detection of Rickettsia in ticks from Sierra Nevada de Santa Marta, Colombia
Source: Exp Appl Acarol. 2024 Mar 14;92(3):507–28. doi: 10.1007/s10493-023-00887-z (PMC11035439; doi:10.1007/s10493-023-00887-z)
Supplement: Supplementary file 1 — Supplementary material 1 (DOCX 2980.2 kb) [file 10493_2023_887_MOESM1_ESM.docx]

New hard tick (Acari: Ixodidae) reports and detection of Rickettsia in ticks from Sierra Nevada de Santa Marta, Colombia

**Supplementary material**


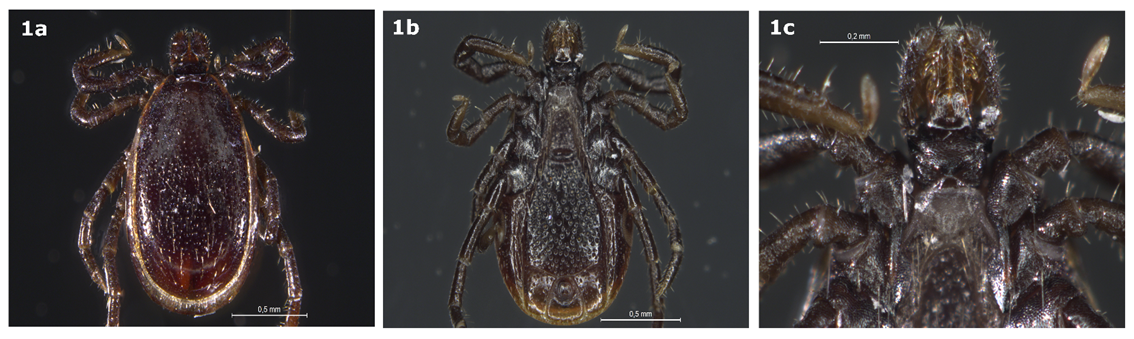


**Figure S1.** Male *Ixodes sp. Cf. affinis.* **(a)** dorsal view, **(b)** ventral view, **(c)** hipostoma ventral view.


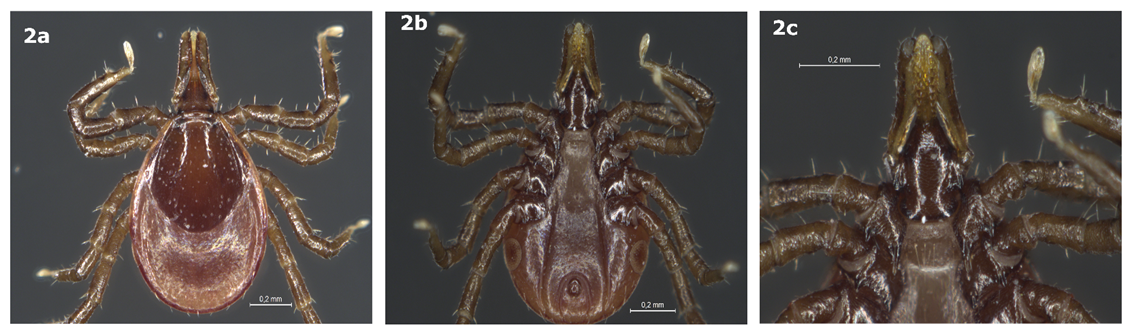


**Figure S2.** Nymph of *Ixodes sp.* **(a)** dorsal view, **(b)** ventral view, **(c)** hipostoma ventral view.


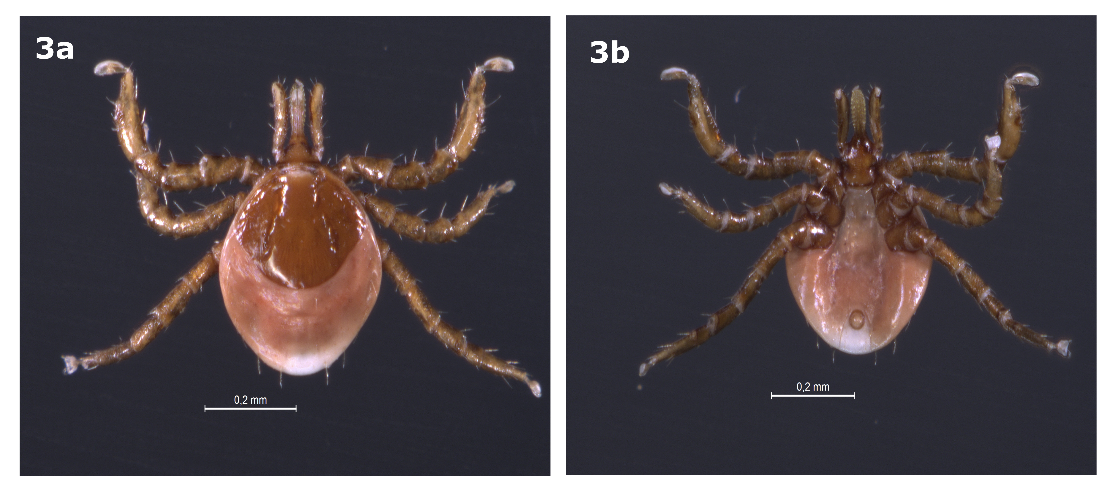


**Figure S3.** Larvae of Ixodes sp. (a) dorsal view, (b) ventral view.


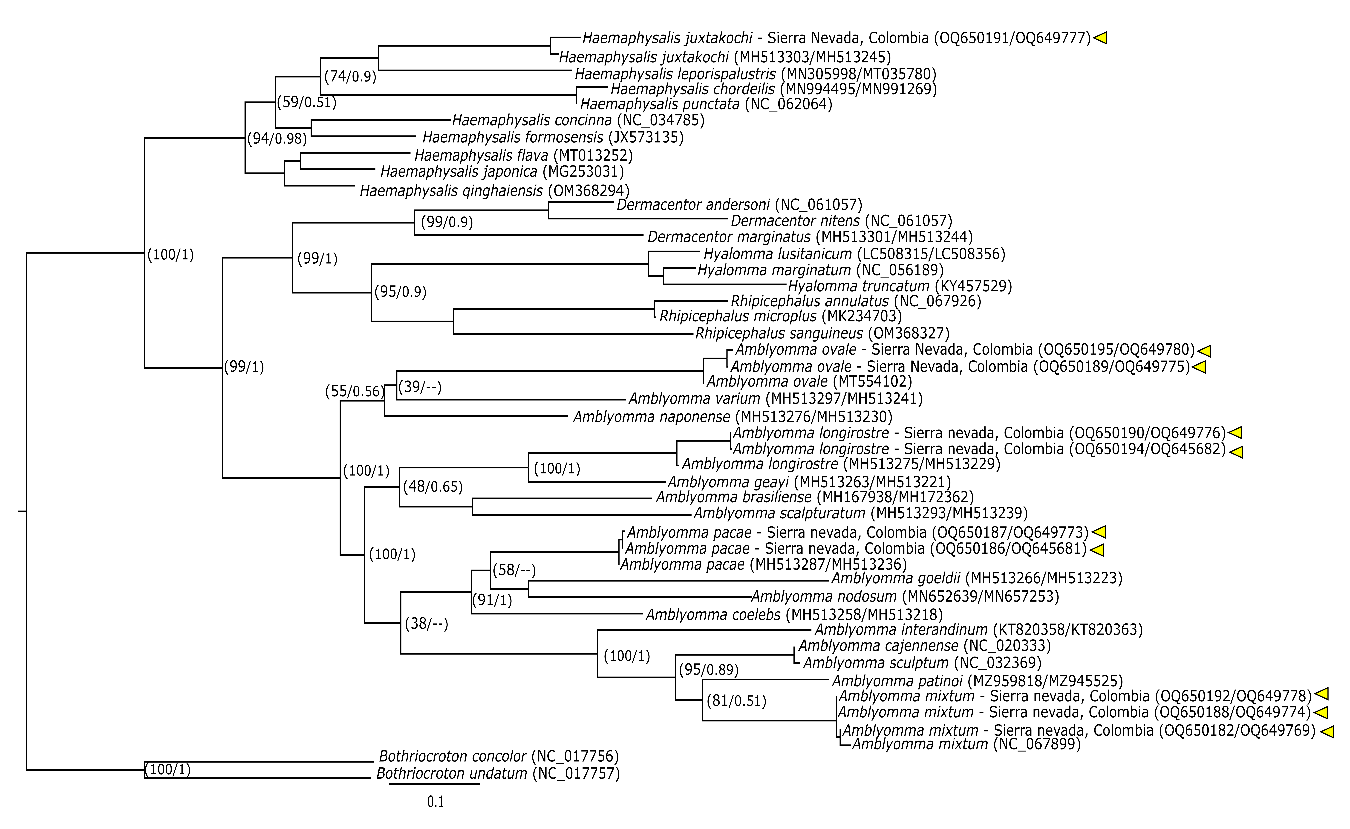


**Figure S4.** Phylogenetic reconstruction using maximum likelihood and Bayesian inference of concatenated *16S* rRNA and *cox1* genes, for our sequences (yellow triangle) and sequences downloaded from GenBank of ticks species of the genus *Amblyomma*, *Haemaphysalis*, Dermacentor, *Hyalomma*, *Rhipicephalus* and *Bothriocroton* (outgroup). Numbers on nodes correspond to bootstrap values/the posterior probability.
